# The relationship between drop vertical jump action‐observation brain activity and kinesiophobia after anterior cruciate ligament reconstruction: A cross‐sectional fMRI study

**DOI:** 10.1002/brb3.2879

**Published:** 2023-01-05

**Authors:** HoWon Kim, James A. Onate, Cody R. Criss, Janet E. Simon, Dominik Mischkowski, Dustin R. Grooms

**Affiliations:** ^1^ Ohio Musculoskeletal & Neurological Institute Ohio University Athens Ohio USA; ^2^ Translational Biomedical Sciences Program, School of Rehabilitation and Communication Sciences, College of Health Sciences and Professions Ohio University Athens Ohio USA; ^3^ Division of Athletic Training, School of Health and Rehabilitation Sciences, College of Medicine Ohio State University Columbus Ohio USA; ^4^ Heritage Fellow, Translational Biomedical Sciences Program, Heritage College of Osteopathic Medicine Ohio University Athens Ohio USA; ^5^ Division of Athletic Training, School of Applied Health Sciences and Wellness, College of Health Sciences and Professions Ohio University Athens Ohio USA; ^6^ Psychology Department, College of Arts and Sciences Ohio University Athens Ohio USA; ^7^ Division of Physical Therapy, School of Rehabilitation and Communication Sciences, College of Health Sciences and Professions Ohio University Athens Ohio USA

**Keywords:** anterior cruciate ligament, kinesiophobia, action‐observation, motor imagery, drop vertical jump, functional magnetic resonance imaging

## Abstract

**Background:**

Injury and reconstruction of anterior cruciate ligament (ACL) result in central nervous system alteration to control the muscles around the knee joint. Most individuals with ACL reconstruction (ACLR) experience kinesiophobia which can prevent them from returning to activity and is associated with negative outcomes after ACLR. However, it is unknown if kinesiophobia alters brain activity after ACL injury.

**Objectives:**

To compare brain activity between an ACLR group and matched uninjured controls during an action‐observation drop vertical jump (AO‐DVJ) paradigm and to explore the association between kinesiophobia and brain activity in the ACLR group.

**Methods:**

This cross‐sectional study enrolled 26 individuals, 13 with ACLR (5 males and 8 females, 20.62 ± 1.93 years, 1.71 ± 0.1 m, 68.42 ± 14.75 kg) and 13 matched uninjured controls (5 males and 8 females, 22.92 ± 3.17 years, 1.74 ± 0.10 m, 70.48 ± 15.38 kg). Individuals were matched on sex and activity level. Participants completed the Tampa Scale of Kinesiophobia‐11 (TSK‐11) to evaluate the level of movement‐related fear. To assay the brain activity associated with a functional movement, the current study employed an action‐observation/motor imagery paradigm during functional magnetic resonance imaging (fMRI).

**Results:**

The ACLR group had lower brain activity in the right ventrolateral prefrontal cortex relative to the uninjured control group. Brain activity of the left cerebellum Crus I and Crus II, the right cerebellum lobule IX, amygdala, middle temporal gyrus, and temporal pole were positively correlated with TSK‐11 scores in the ACLR group.

**Conclusion:**

Brain activity for the AO‐DVJ paradigm was different between the ACLR group and uninjured controls. Secondly, in participants with ACLR, there was a positive relationship between TSK‐11 scores and activity in brain areas engaged in fear and cognitive processes during the AO‐DVJ paradigm.

## Graphical abstract

Brain activity for the action‐observation drop vertical jump paradigm was different between the ACLR group and uninjured controls. Secondly, in participants with ACLR, there was a positive relationship between TSK‐11 scores and activity in brain areas engaged in fear and cognitive processes during the action‐observation drop vertical jump paradigm.

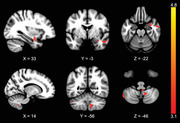

## 1 INTRODUCTION

Anterior cruciate ligament (ACL) injury is a common knee injury in physically active populations (Kaeding et al., 2017; Musahl & Karlsson, 2019; Spindler & Wright, 2008). In the United States, reconstructive surgery is the standard of care after ACL injury to restore mechanical stability of the knee joint (Carey et al., 2009). Following reconstruction surgery, individuals typically participate in structured rehabilitation to regain functional performance and return to preinjury activity levels. However, even after 6–9 months of rehabilitation (Panariello et al., 2017), outcomes after ACL reconstruction (ACLR) are not favorable. Previous literature has documented that individuals with ACLR have a higher risk for early onset posttraumatic knee osteoarthritis compared to uninjured populations (Watters et al., 2017), and 25% will go on to sustain a second ACL injury after returning to activity (Paterno et al., 2012; Zacharias et al., 2021). Several factors have been postulated to contribute to the increased secondary injury and posttraumatic knee osteoarthritis risk, including prolonged quadriceps muscle strength deficits (Hart et al., 2010; Ingersoll et al., 2008; Myer et al., 2012) and altered biomechanics during physical activities, such as walking and double leg jump‐landing (Erhart‐Hledik et al., 2018; Goerger et al., 2015). However, there is limited evidence that explains the underlying mechanisms for why individuals with ACLR experience prolonged deficits even after a knee‐focused rehabilitation program.

Previous research has suggested that the central nervous system (CNS) alterations following ACLR might contribute to prolonged knee‐related functional deficits (Chaput et al., 2022; Criss et al., 2020; Grooms et al., 2017; Lepley et al., 2015; Pietrosimone et al., 2015). The CNS alterations may be initiated by the disruption of mechanoreceptors in the ACL, which are sensory receptors that send afferent proprioceptive information to the brain. Proprioception provides crucial sensory information to maintain appropriate knee joint neuromuscular control and avoid injury risk loading (Kennedy et al., 1982). However, individuals with a history of ACLR experience prolonged sensory deafferentation that is not restored with surgery (Valeriani et al., 1999). In addition to the sensory deafferentation after ACL injury, rehabilitation strategies might further affect CNS plasticity and contribute to prolonged deficits in neuromuscular control (An et al., 2019; Criss et al., 2020; Grooms et al., 2017; Lepley et al., 2015).

In addition to the biomechanical and neurophysiological implications, individuals with ACLR experience psychological alterations, such as kinesiophobia (fear of movement/re‐injury) (Lentz et al., 2014; Paterno et al., 2018). The Tampa Scale of Kinesiophobia‐11 (TSK‐11) is a common patient‐reported outcome measure to identify kinesiophobia in individuals with a history of ACL injury (Dashti Rostami et al., 2021; Meierbachtol et al., 2020; Paterno et al., 2018; Tajdini et al., 2021; Trigsted et al., 2018). Over half (61.69%) of individuals, 4–8 weeks after ACLR, report elevated levels of kinesiophobia (Chaitanya Shah et al., 2017), which does not fully recover when they return to activity (>47.5%) (Dashti Rostami et al., 2021; Paterno et al., 2018). Having an elevated TSK‐11 score not only prevents individuals from returning to activity (Flanigan et al., 2013), it also contributes to a higher probability of asymmetric quadriceps strength, hop distance, and vertical ground reaction force during walking and landing (Dashti Rostami et al., 2021; Paterno et al., 2018; Tajdini et al., 2021). Furthermore, a TSK‐11 score greater than 18 is associated with increased risk of secondary ACL injury (Paterno et al., 2018). However, there is limited evidence about the relationship between kinesiophobia and brain activity after ACLR which may be the physiologic pathway that reduces functional performance (Paterno et al., 2018; Tajdini et al., 2021; Trigsted et al., 2018).

Functional magnetic resonance imaging (fMRI) is one of the standard methods to indirectly measure neuronal activity during cognitive and physical tasks, and provides relatively high spatial and temporal resolution (Logothetis, 2008). Previous neurophysiologic studies have provided fundamental lower extremity brain activation patterns while performing simple uniplanar movements (knee or hip flexion and extension while lying supine) or muscle contractions after ACL injury (Chaput et al., 2022; Criss et al., 2020; Grooms et al., 2017; Kapreli & Athanasopoulos, 2006; Lepley et al., 2019). However, due to primarily technical limitations, these methods did not replicate injury‐relevant movements. A way to circumvent the technical limitations and assay brain activity associated with more injury‐relevant movement patterns is to take advantage of the mirror neuron system (MNS) (Rizzolatti & Sinigaglia, 2016). The MNS represents the brain activation pattern that engages in a movement execution during the imagination or visual immersion of the movement (Hyun et al., 2015; Taube et al., 2015). Due to this similarity in activation pattern, it is possible to use the MNS to probe the brain activation strategy for more complex movements while adhering to the technical restraints required for neuroimaging (supine positioning, minimal head motion, etc.) (di Pellegrino et al., 1992; Hardwick et al., 2018; Hyun et al., 2015; Rizzolatti & Sinigaglia, 2016; Taube et al., 2015). Therefore, the use of an action‐observation technique with motor imagery can provide a means to address the gap between experimental movement designs and more functional injury‐relevant movement patterns.

The primary purpose of the current study was to compare brain activity between individuals with ACLR and uninjured controls during an action‐observation drop vertical jump (AO‐DVJ) paradigm. The secondary purpose was to determine the neural correlates between kinesiophobia and brain activity during the AO‐DVJ paradigm in those with ACLR.

## 2 METHODS

### 2.1 Participants

The current study used a subset of participants from a large cross‐sectional study that examined various aspects of strength, balance, biomechanics, functional performance, and brain activity during several movement and action‐observation motor imagery paradigms (Grooms et al., 2017). Thus, this is a secondary data analysis of the larger study and a subset of participants who completed the AO‐DVJ paradigm were selected for the current analysis, a formal sample size calculation was not conducted. Twenty‐six participants, 13 individuals with a history of left ACLR (5 males and 8 females) and 13 uninjured controls (5 males and 8 females) from local colleges and orthopedic clinics were included in the current study (Table 1). The ACLR group was 1–10 years postsurgery and cleared to return to activity by their physician. The ACLR group was required to meet the following criteria: isolated ACL injury on left knee, Tegner activity score higher than 5 at the time of participation (participating in recreational sports at least 5 times per week), intended to return to their previous activity level, no other lower extremity injury within a year, and aged between 18–35 years. The uninjured controls were required to meet all the above criteria other than the history of ACL injury. The uninjured control participants reported no history of lower extremity injury or surgery and were matched to ACLR individuals with regard to sex and physical activity level. Participants were excluded if they had a history of neurological disorder or any contraindication to fMRI. This study was approved by the Ohio State University Institutional Review Board (IRB), and all participants volunteered to enroll and signed an informed consent form prior to participation.

**TABLE 1:** Descriptive statistics of demographics and patient‐report outcome measures

	ACLR	Uninjured control	*p* Value	Effect size
Sex (F:M)	8:5	8:5	–	–
Age (years)^+^	20 (19–24)	22 (19–29)	.04*	0.40
Height (m)	1.71 ± 0.10	1.74 ± 0.10	.42	0.32
Weight (kg)	68.42 ± 14.75	70.48 ± 15.38	.73	0.14
BMI	23.02 ± 2.94	22.79 ± 2.87	.84	0.08
Tegner^+^	7 (6–10)	7 (6–9)	.73	0.08
TSK‐11	17.85 ± 3.95 (*n* = 13)	13.45 ± 2.46 (*n* = 11)	.01*	1.31
IVI^+^	5.5 (4.75–7)	5.5 (5–6.75)	.39	0.18
EVI	5.77 ± 0.70	5.98 ± 0.83	.49	0.28
KI	5.56 ± 0.85	4.87 ± 1.00	.07	0.75

*Note*: Height, weight, BMI, TSK‐11, EVI, and KI were reported as mean ± standard deviation. Age, Tegner activity score, and IVI were reported as median (range). Effect size: Cohen's d for parametric tests & *r* = |*z*|/√*n* for nonparametric tests.

* Significantly different *p* < .05.

^+^ Nonparametric test (Mann‐Whitney *U* test).

BMI: body mass index, Tegner: Tegner activity score, TSK‐11: Tampa Scale for Kinesiophobia 11, IVI: internal visual imagery score, EVI: external visual imagery score, KI: kinesthetic imagery score.

### 2.2 Patient‐reported outcome measures

All participants completed the Tegner activity score, Movement Imagery Questionnaire‐3 (MIQ3), and the TSK‐11 prior to the fMRI session to evaluate current physical activity level, motor imagery skills, and kinesiophobia, respectively. The Tegner activity score consists of one question which indicates patient‐reported activity level (Tegner, 1985). The rating is from 0 to 10, with a higher rating indicating a higher level of activity.

The MIQ3 is a valid and reliable measure of visual and kinesthetic movement imagery ability and consists of three subscales: internal visual imagery (IVI), external visual imagery (EVI), and kinesthetic imagery (KI) (Williams et al., 2012). It consists of 12 questions, with each question rated from 1–7. A rating of 1 indicates very hard to see∖feel the movement and a rating of 7 indicates very easy to see∖feel the movement, with a higher score indicating a higher ability to perform the imagery techniques. Participants were told to imagine performing 4 different movements with each imagery technique: hip hike, bend over, shoulder horizontal adduction, and vertical jump.

The TSK‐11 is a shortened version of the TSK‐17 where six questions were eliminated due to poor psychometric performance. The TSK‐11 is a reliable measurement to identify movement‐related fear and/or fear of re‐injury (French et al., 2007; Swinkels‐Meewisse et al., 2003; Woby et al., 2005). The TSK‐11 consists of 11 questions that asks individuals about perceptions of fear related to pain and re‐injury based on movements (Kinisophobia , 1990; Woby et al., 2005). Each question is measured by scores on a 4‐point scale: strongly disagree (1 point) to strongly agree (4 points). Total scores range from 11–44 points, with higher scores indicating increased fear. TSK‐11 scores were averaged and demeaned for each group. Demeaned TSK‐11 scores were used as a covariate for the neuro‐correlates analyses to investigate the relationship between TSK‐11 scores and brain activity.

### 2.3 Neuroimaging data collection

Three‐dimensional high‐resolution T1‐weighted images (repetition time [TR]: 1950 ms, echo time [TE]: 4.44 ms, field of view [FOV]: 256 × 256 mm; matrix: 256 × 256; slice thickness: 1 mm; and 176 slices) were collected on a 3T Siemens Magnetom scanner with a 12‐channel head coil prior to functional neuroimaging. Functional neuroimaging data were collected on the same MRI machine (TR: 3000 ms; TE: 28 ms; FOV: 220 × 220 mm; flip angle: 78; slice thickness: 2.5 mm; 55 transversal slices; voxel size: 2.5 mm^3^; and interleaved slice timing).

**FIGURE 1:**
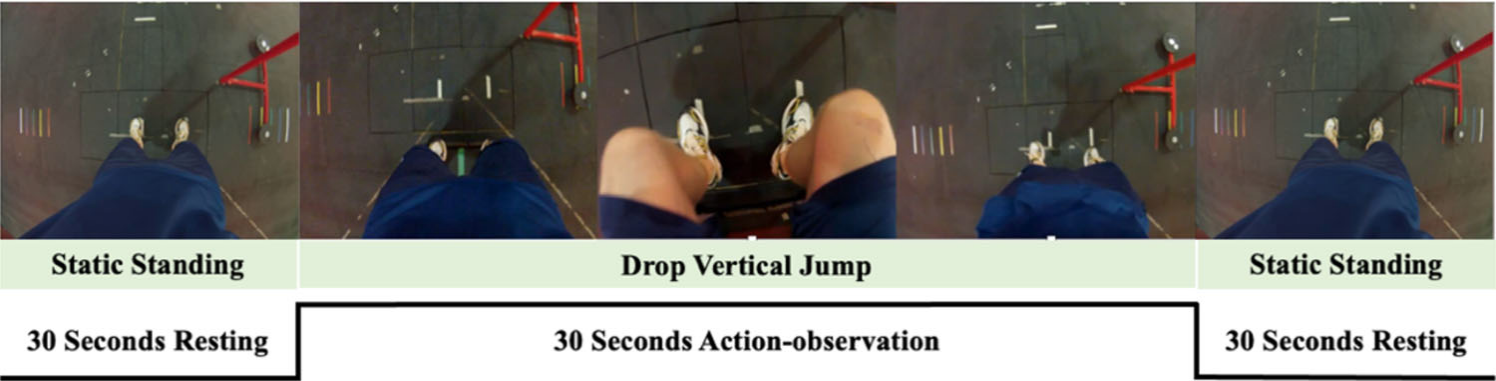
Action‐observation drop vertical jump paradigm

All participants were positioned supine on the MRI table and straps were secured over their chest and anterior superior iliac spines to prevent accessory movement. Participants wore headphones and hearing protection for comfort and safety. A mirror was attached to the top of the head coil that reflected a monitor behind the MRI machine for observing the AO‐DVJ paradigm. A 15‐min T1 structural scan was obtained prior to functional neuroimaging data collection with the AO‐DVJ paradigm. The fMRI AO‐DVJ paradigm consisted of five blocks of 30‐s rest and four blocks of 30‐s task conditions between resting conditions. The current paradigm was developed based on previous studies that evaluated brain activity during action‐observation, motor imagery, and/or motor execution tasks (la Fougère et al., 2010; Miyai et al., 2001; Sacheli et al., 2018; Wang et al., 2008). During the task blocks the participants observed 12 consecutive drop vertical jumps that were displayed from a first‐person perspective. Participants were instructed to imagine themselves as a person in the monitor, and they were executing the observed task. The drop vertical jump was used during the AO‐DVJ paradigm due to the prospective association with primary (Hewett et al., 2005; Leppänen et al., 2017) and secondary ACL injury (Paterno et al., 2010), being cited as a common fear‐inducing movement (Meierbachtol et al., 2020), and complying with fMRI limitations for ease of immersion (minimizing perspective shifts to reduce motion sickness). During the resting blocks, participants watched a static standing image in a first‐person perspective (Figure 1).

### 2.4 Statistical analysis

Descriptive statistics were calculated for demographics and patient‐reported outcome measures using SPSS version 27 (SPSS Inc., Chicago, IL). The demographics and patient‐reported outcome measures were assessed for the assumptions of normality and homogeneity of variances using Shapiro‐Wilk and Levene's tests, respectively. Age, Tegner, and IVI violated the assumption of normality thus, nonparametric tests were used. Independent *t*‐tests were used to determine whether there were statistically significant mean differences between the ACLR and uninjured control groups in demographics (height, weight, and BMI) and patient‐reported outcome measures (TSK‐11, EVI, and KI) that did not violate assumptions *(p <* .05). Mann‐Whitney U tests were used to determine whether there were statistically significant median differences for age, Tegner activity score, and IVI between the ACLR and uninjured control groups *(p <* .05). Data were presented as mean ± standard deviation or median with range for parametric and nonparametric tests, respectively.

#### 2.4.1 fMRI statistical analyses

The fMRI data were preprocessed using the software package Oxford Centre for Functional MRI of the Brain Software Library (FSL) 6.00 (FMRIB, Oxford UK). All analyses underwent standardized processing which included brain extraction, MCFLIRT motion correction, interleaved slice timing correction, intensity normalization, and 6 mm spatial smoothing based on Gaussian's random field theory (Jenkinson et al., 2002; Smith, 2002; Smith et al., 2004). An Independent Component Analysis‐based strategy for the Automatic Removal of Motion Artifacts (ICA‐AROMA) was used to denoise and reduce motion‐induced signal variations within the data. Following ICA‐AROMA, a high pass filter at 100 s was applied to the data. Functional neuroimaging data were linearly registered to structural (T1‐weighted image) and nonlinearly registered to standard (Montreal Neurological Institute [MNI] 152, 2 mm brain) spaces.

First‐level analysis was completed for all participants to contrast between two conditions: condition 1 (rest) and condition 2 (task [AO‐DVJ]). For all the neuroimaging analyses, a priori threshold was set at *z* = 3.1 and an alpha level of .05 with a cluster‐corrected significance threshold for multiple comparisons.

To determine average brain activity during the AO‐DVJ paradigm from all participants, a general linear model (GLM) second‐level mixed‐effect analysis was conducted. Full model analysis was set up as a GLM with one explanatory variable: group. The GLM was set up with one contrast, group mean. Brain regions were defined in all statistical clusters as representing greater than 1.0 probabilistic threshold from the *atlasquery* command in FSL and greater than 10 voxels of the anatomical region present in the cluster.

For comparing brain activity between the ACLR and matched uninjured control groups during the AO‐DVJ paradigm, a GLM second‐level mixed‐effects paired analysis was performed. Full model analysis was set up as a GLM with one explanatory variable: group. The GLM was set up with two contrasts, higher relative activity in the ACLR group and higher relative activity in the uninjured control group.

To determine the correlations between TSK‐11 scores and gray matter brain activity during the AO‐DVJ paradigm in the ACLR group, a GLM second‐level mixed‐effects analysis was conducted. Full model analysis was set up as a GLM with two explanatory variables, group and demeaned TSK‐11 scores. The GLM was set up with three contrasts, group mean, TSK‐11 (positive correlation), and TSK‐11‐reverse (negative correlation). To investigate brain activity only in the gray matter, a gray matter mask was used as a prethreshold mask that generated by FMRIB's automated segmentation tool (FAST) and averaged across subjects (Zhang et al., 2001).

## 3 RESULTS

### 3.1 Demographics and patient‐reported outcome measures

Demographic information is provided in Table 1. Two uninjured control participants did not complete the TSK‐11 questionnaire and were excluded from the neuro‐correlates analysis. The TSK‐11 score was significantly higher in the ACLR group (17.85 ± 3.95 [*n* = 13]) compared to the uninjured control group (13.45 ± 2.46 [*n* = 11], *t*_(22)_ = 3.19, *p* = .004, Cohen's *d* = 1.31). Age was lower in the ACLR group (20 [19–24]) compared to the uninjured control group (22 [19–29], *U*_(24)_ = 123, *p* = .044, effect size = 0.40). Nine participants in the ACLR group (69%) and two participants in the uninjured control group (18%) had a score greater than 16 on the TSK‐11, indicating high fear (Paterno et al., 2018). There were no significant differences for any other demographics or patient‐reported outcome measures between the ACLR and uninjured control groups. However, there was a large effect size in KI score. The higher KI score in the ACLR group was driven by movements that were not related to jumping or knee joint movements (Supplementary Table S1) and thus, was not relevant to the AO‐DVJ paradigm.

### 3.2 Neuroimaging

**FIGURE 2:**
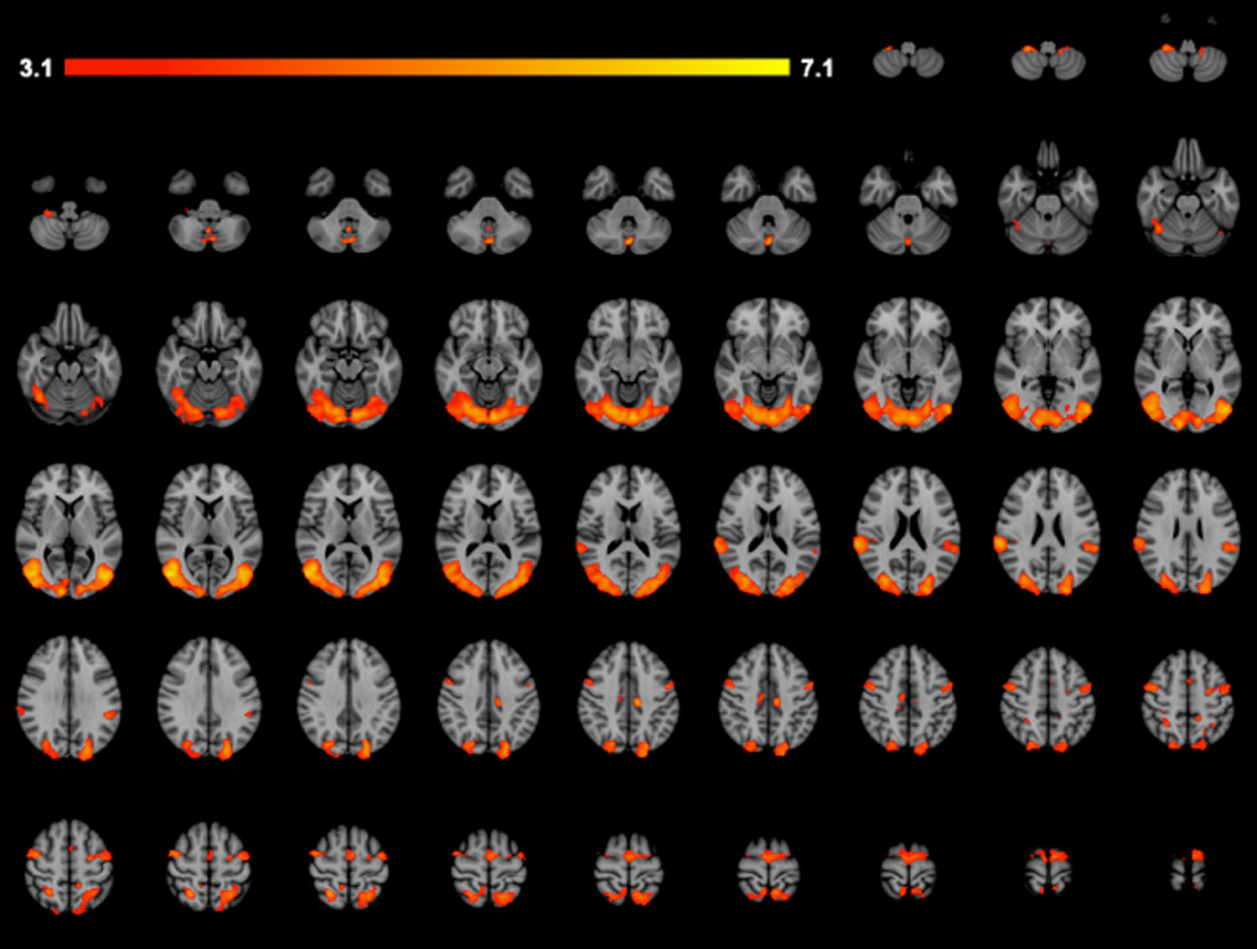
Average brain activation during the action‐observation drop vertical jump paradigm

Average brain activation of all participants (26 total) during the AO‐DVJ paradigm showed activation in the sensorimotor area, visual cortex, and cerebellum (Figure 2; Table 2).

**TABLE 2:** Regions of brain activity during the action‐observation drop vertical jump paradigm

				Peak MNI* voxel			
Cluster index	Brain regions	Voxel	*p* Value	*x*	*y*	*z*	*Z* max
1	Left cerebellum	126	.05	–16	–42	–52	4.61
	Lobule VIIIa						
	Lobule VIIIb						
	Lobule IX						
	Lobule X						
2	Left	185	.008	–10	–22	42	5.82
	Primary motor cortex						
	Superior parietal lobule						
	Supplementary motor cortex						
	Premotor cortex						
	Cingulate gyrus						
3	Right	198	.006	8	–16	46	4.71
	Primary motor cortex						
	Primary sensory cortex						
	Superior parietal lobule						
	Supplementary motor cortex						
	Premotor cortex						
	Cingulate gyrus						
4	Right cerebellum	279	.0007	28	–40	–52	5.91
	Lobule VIIb						
	Lobule VIIIa						
	Lobule VIIIb						
	Lobule IX						
	Lobule X						
5	Left	522	<.0001	–58	–40	24	5.42
	Inferior parietal lobule						
	Secondary sensory cortex						
	Superior temporal gyrus						
6	Right	818	<.0001	66	–34	22	6.29
	Inferior parietal lobule						
	Secondary sensory cortex						
	Superior temporal gyrus						
7	Bilateral	3029	<.0001	44	–2	60	5.66
	Premotor cortex						
	Supplementary motor cortex						
	Left						
	Primary motor cortex						
8	Bilateral	19509	<.0001	54	–72	8	7.1
	Superior parietal lobule						
	Visual cortex						
	Precuneus						
	Lingual gyrus						
	Optic radiation						

* MNI: Montreal Neurological Institute. Harvard‐Oxford cortical structural atlas, Juelich histological atlas, and Cerebellar atlas in MNI 152 space after normalization with FNIRT were used to determine the anatomical areas.

**FIGURE 3:**
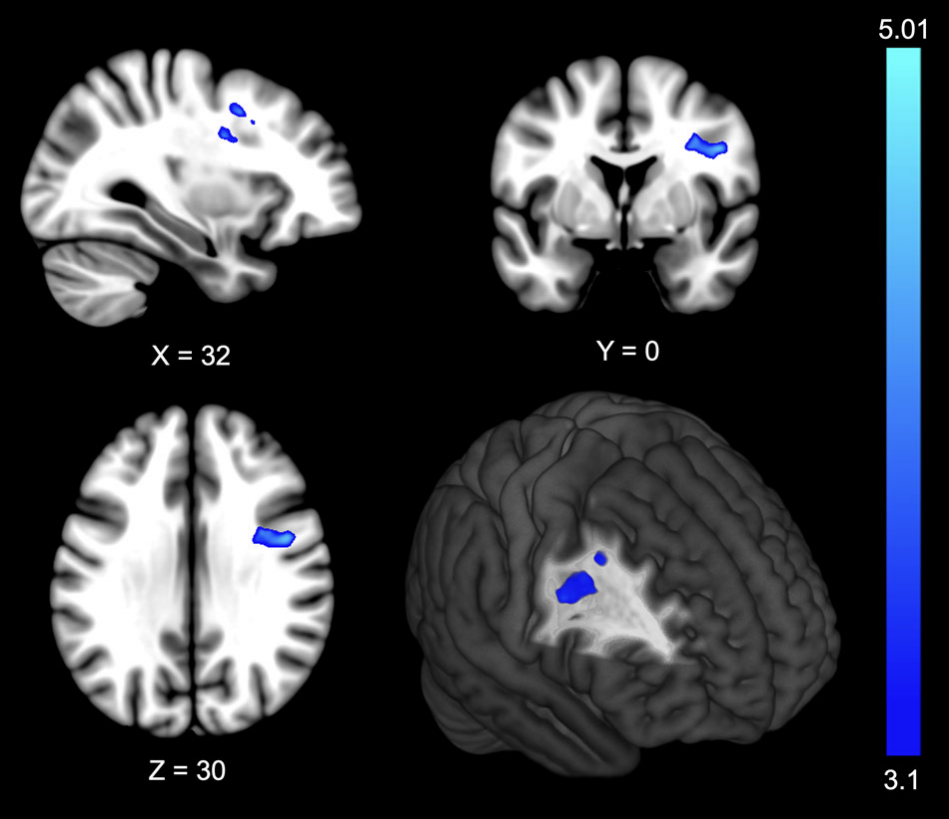
Lower brain activity during the action‐observation drop vertical jump paradigm in the ACLR group

The ACLR group had lower brain activity in the right ventrolateral frontal cortex including the precentral gyrus, middle frontal gyrus, and corticospinal tract relative to the uninjured control group for the AO‐DVJ paradigm (Figure 3; Table 3). There was no brain region with greater activity in the ACLR group compared to the uninjured control group. Group comparison brain activity results were similar between those with and without two high fear uninjured control and four low fear ACLR participants (Supplementary Figure S1).

**TABLE 3:** Brain regions that demonstrated lower activation in the ACLR group relative to the uninjured control group

					Peak MNI* voxel			
Cluster index	Brain regions	Anatomical region voxels	Cluster voxels	*p* Value	*x*	*y*	*z*	*Z* max
1	Right		267	.0003	32	0	30	4.77
	Precentral gyrus	75						
	Middle frontal gyrus	25						
	Corticospinal tract	39						

* MNI: Montreal Neurological Institute. Harvard‐Oxford cortical structural atlas and Juelich histological atlas were used to determine the anatomical areas.

**FIGURE 4:**
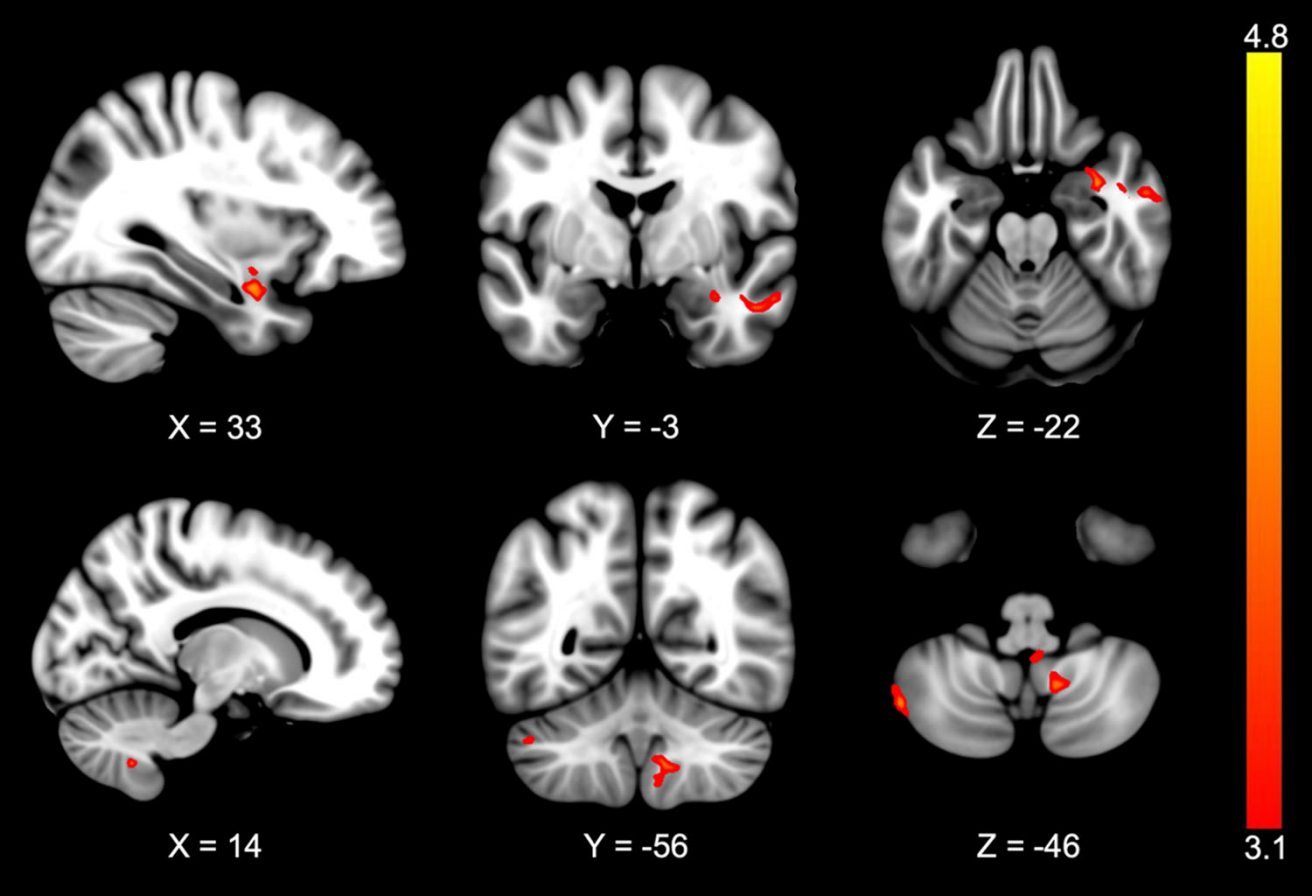
Brain activity positively correlated with TSK‐11 scores during the action‐observation drop vertical jump paradigm in the ACLR group

Brain activity of the left cerebellum Crus I and Crus II, the right cerebellum lobule IX, amygdala, middle temporal gyrus, and temporal pole were positively correlated with TSK‐11 scores in the ACLR group (Figure 4; Table 4). There were no negative correlations between brain activity and TSK‐11 scores in the ACLR group. There were no correlations between brain activity and TSK‐11 scores in the uninjured control group.

**TABLE 4:** Regions of brain activity that positively correlated with TSK‐11 scores during the action‐observation drop vertical jump paradigm in the ACLR group

					Peak MNI* voxel			
Cluster index	Brain regions	Anatomical region voxels	Cluster voxels	*p* Value	*x*	*y*	*z*	*Z* max
1	Left		115	.043	–52	–60	–46	4.41
	Crus I	59						
	Crus II	52						
2	Right		116	.042	14	–56	–46	4.43
	Lobule IX	92						
3	Right		177	.006	34	0	–20	4.59
	Amygdala	26						
	Temporal pole	10						
	Middle temporal gyrus	88						
4	Right		185	.005	50	14	–38	4.80
	Temporal pole	152						
	Middle temporal gyrus	16						

* MNI: Montreal Neurological Institute. Cerebellar atlas in MNI 152 space after normalization with FNIRT, Harvard‐Oxford cortical structural atlas, and Harvard‐Oxford Subcortical structural atlas.

## 4 DISCUSSION

The ACLR group had lower brain activity in the right ventrolateral prefrontal region compared to the uninjured controls during the AO‐DVJ paradigm. Additionally, higher TSK‐11 scores were associated with greater brain activation in the left cerebellum Crus I and Crus II, the right cerebellum lobule IX, amygdala, middle temporal gyrus, and temporal pole during the AO‐DVJ paradigm only in the ACLR group.

### 4.1 Brain activation during the action‐observation drop vertical jump paradigm

In the current study, average brain activity from all the participants for the AO‐DVJ paradigm was computed (Figure 2; Table 2) to compare to brain activity patterns found in previous studies. Sensorimotor regions, visual cortex, and cerebellum were activated during the AO‐DVJ paradigm, which have also been activated during lower extremity motor execution and gait motor imagery tasks (Allali et al., 2014; Criss et al., 2020; la Fougère et al., 2010; Newton et al., 2008; van der Meulen et al., 2014). A key difference between our study and prior motor execution studies was the lack of primary motor cortex activity which is to be expected with an action‐observation and motor imagery paradigm.

The AO‐DVJ paradigm used in the current study included both action‐observation and motor imagery techniques. Based on the MNS (di Pellegrino et al., 1992), both techniques are well accepted modalities that induce brain activity patterns relative to actual motor execution (Hardwick et al., 2018). The MNS was first discovered in a macaque monkey brain (di Pellegrino et al., 1992); however, recent findings have indicated the presence of the MNS in the human brain located mostly in the inferior parietal lobule, ventral premotor cortex, posterior inferior frontal gyrus, superior temporal sulcus, primary motor cortex, and supplementary motor area (Caspers et al., 2010; Hardwick et al., 2018).

Activation of the MNS is based on the relationship between perception of movement and the internal model to imitate observed movement (Kilner et al., 2007; Massen & Prinz, 2009; Prinz, 1997). Perception of movement is an internal process that helps a person to understand and predict the result of the observed movement based on the internal model (Kilner et al., 2007). The internal model is an inherent motor program that is learned and updated from previous experiences that helps the CNS to correctly predict the consequence of motor commands (Shadmehr & Krakauer, 2008). These internal processes are also involved in the motor planning stage of movement (Haggard et al., 2008; Prinz, 1997). Therefore, the group average brain activation patterns during the AO‐DVJ paradigm may represent the motor planning process of the drop vertical jump task, and these patterns may indicate that the participants were mentally engaged in the task.

### 4.2 Group differences in brain activity

The ACLR group had lower brain activity in the right ventrolateral prefrontal region compared to the uninjured controls during the AO‐DVJ paradigm. The statistical cluster of lower activity encompasses primarily the ventrolateral prefrontal cortex but also consisted of the precentral gyrus, middle frontal gyrus, and corticospinal tract.

#### 4.2.1 Altered brain activity for action representation and prediction

The ventrolateral prefrontal cortex, specifically the middle frontal cortex, plays a role in reorienting attention from a bottom‐up to a top‐down attentional system (Japee et al., 2015). The top‐down system is based on the dorsal attention network to accomplish specific goals and prepare motor responses (Corbetta et al., 2008; Corbetta et al., 2000; Rushworth et al., 2001). Engagement of the dorsal attentional network may help participants to focus on crucial information from the AO‐DVJ paradigm to understand the purpose of the movement and imitate the motor command. Lower brain activity in the ventrolateral prefrontal cortex may be a contributing brain mechanism in those with a history of ACLR who have decreased ability to switch attention during cognitive‐motor dual‐tasks and/or unexpected perturbations relative to uninjured controls (Heidarnia et al., 2022; Smeets et al., 2021). Decreased ability to reorientate attention between imperative and extraneous information to maintain motor control may result in inaccurate predictions of movement, leading to motor planning errors.

Furthermore, the ventral premotor cortex is crucial for visual discrimination of actions, and impairment of the ventral premotor region can cause slower reaction time for action discrimination processing (Urgesi et al., 2007). Thus, the lower brain activity in the ventral premotor cortex for the ACLR group may explain prior findings of increased motor planning or perceptual judgment processing time after ACLR (Armitano‐Lago et al., 2020). Such delays in a dynamic sports environment may reduce a person's ability to maintain knee neuromuscular control when perceptual attention is constantly distracted within the environment.

#### 4.2.2 Interpretation relative to prior studies

Previous fMRI research has shown higher activity in the visual cortex during a simple knee movement task in an ACLR group compared to uninjured controls (Grooms et al., 2017). This result indicates a potential visual reliance strategy (or related cross‐modal or visual‐cognitive processing) for knee neuromuscular control due to sensory reweighting as a compensation for deafferentation after ACLR (Criss et al., 2020; Grooms et al., 2017). The prolonged engagement of such a strategy may result in neural efficiency or lower brain activity to utilize visual information to activate the MNS related to knee movements in the ACLR group. As the ventrolateral prefrontal region integrates spatial (orientation, position, velocity, and depth) and objective (perception: shape, color, texture) visual information, lower brain activity in this region might be the result of neural efficiency (Takahashi et al., 2013). However, this visual strategy might not be ideal in a real‐life situation, such as walking on a busy street or in sports environments when vision is occupied by the environment (Aksum et al., 2021).

### 4.3 Kinesiophobia neural correlates in the ACLR group

In the ACLR group, a higher level of kinesiophobia was associated with greater brain activity in the left cerebellum Crus I and Crus II, the right cerebellum lobule IX, amygdala, middle temporal gyrus, and temporal pole during the AO‐DVJ paradigm.

Activities in the amygdala and middle temporal gyrus are associated with fearful or potentially pain‐inducing events (Meier et al., 2016; Schäfer et al., 2005; Schienle et al., 2005). Such a brain activation pattern associated with elevated TSK‐11 scores in the ACLR group supports a potential fear response during the AO‐DVJ paradigm. Increased activity in the amygdala has been shown in previous research in individuals with low back pain viewing pain‐inducing movements, individuals with arachnophobia viewing spiders, or uninjured people viewing fearful events (Meier et al., 2016; Schäfer et al., 2005; Schienle et al., 2005). However, it is unlikely that the response seen in the current study is at the same level as those with prolonged and debilitating chronic pain or arachnophobia. Individuals with chronic low back pain report higher levels of kinesiophobia compared to the ACLR group in this study (Meier et al., 2016) and had increased activity in additional brain regions related to fear, such as the orbitofrontal cortex, insula, and parahippocampal gyrus (Meier et al., 2016; Schienle et al., 2005). Also, the experimental design in the current study did not specifically induce fear, nor the level of fear reported during the AO‐DVJ paradigm. However, neural correlates of TSK‐11 scores in the ACLR group indicate that those with elevated level of kinesiophobia may have a unique neural response to imagined or observed drop vertical jumps.

The Crus I and II of the cerebellum are involved in cognitive processes and Crus I activity is also associated with aversive stimuli (Moulton et al., 2011; Stoodley & Schmahmann, 2009). In addition to the amygdala and temporal gyrus activity, increased activity in the Crus I may indicate that the ACLR group with elevated TSK‐11 scores may consider the drop vertical jump as a fearful or adverse event. Since the AO‐DVJ paradigm may provide brain activity corollary to motor planning, the altered brain activity patterns may represent a unique neural mechanism in those with high fear while planning to perform a drop vertical jump. This unique activation strategy may characterize the neural mechanism for stiffer landing strategies (decreased sagittal knee, hip, and trunk motion and increased frontal knee movement) in ACLR groups with elevated kinesiophobia (Trigsted et al., 2018). The stiffer landing neural strategy hypothesis may have additional support with increased activity in the cerebellar lobule IX. The lobule IX is associated with lower extremity movements (Mottolese et al., 2013; Xiong & Matsushita, 2000) and has a connection with the vestibular nuclei, which engages in postural alignment and controls of antigravity muscles (Markham, 1987). Further, as elevated TSK‐11 scores increase the risk of secondary ACL injury (Trigsted et al., 2018), it is possible that neural activity associated with the TSK‐11 score plays a role in noncontact motor coordination errors that lead to injury (Swanik, 2015).

The neural activity contributing to motor coordination errors in those with ACLR and high TSK‐11 scores is supported by prior work in injured gymnasts (Calmels et al., 2014). Injured gymnasts also had greater Crus I activity during an action‐observation of gymnastic movements when they were injured and unable to perform the movements relative to when they were recovered. The authors attributed the increased Crus I activity to the injury and prolonged inability to practice the movements leading to reduced acuity of sensory predictions and matching of motor consequences (greater error between sensory prediction and motor plan vs. movement outcome and associated feedback) (Calmels et al., 2014). A potential extrapolation between the study in gymnasts to the current study is that elevated Crus I activity in those with ACLR and high TSK‐11 scores may increase probability of coordination errors and elevated second injury risk (Trigsted et al., 2018), due to subtle changes in the internal model to predict sensory responses from movement (Calmels et al., 2014).

## 5 LIMITATIONS

This study was a secondary data analysis from a larger cross‐sectional study. Thus, a power analysis was not conducted, and future research should repeat this study on a new sample. The uninjured control participants were matched to the ACLR group for sex and Tegner activity score, but the uninjured control group was significantly older (∼2 years) than the ACLR group. However, most participants were in their early 20s (five participants were 19 years old), so it is unlikely the 2 years of difference (ACLR: 20 [19–24] vs. uninjured control: 22 [19–29] years) influenced the results (Giedd et al., 1999). Also, we did not control for sex, even though sex might impact long‐term outcomes following an ACL injury (Sánchez Romero et al., 2021).

We only measured brain activity during the AO‐DVJ paradigm. Owing to an absence of proprioceptive information, brain activity from the current study might not predict brain activity while performing an actual drop vertical jump task. In addition, the TSK‐11 is designed to measure fear related to general movement. Having a measure to assess fear during a drop vertical jump task would provide a better understanding of the results from our neural correlates analyses. Additionally, even though the AO‐DVJ paradigm was used as it has injury relevance, other tasks may elicit a greater prevalence of kinesiophobia (Meierbachtol et al., 2020).

## 6 FUTURE RESEARCH DIRECTIONS

The current study found a similar brain activation pattern between the AO‐DVJ paradigm and previous manuscripts on motor execution (Criss et al., 2020; Newton et al., 2008). However, it would be beneficial to conduct future studies to compare brain activity between the action‐observation with motor imagery paradigm and actual motor execution in an ACLR group, such as brain activity during a bilateral hip‐knee flexion/extension movement (a similar movement as the drop vertical jump). Also, to provide a fully immersive experience during the action‐observation paradigm, future research should consider using a 360° video clip presented through a virtual reality headset to compare with the current study. Furthermore, a third‐person perspective with the same movement to examine the ACLR group's ability to predict another person's movement may be beneficial to examine the possible mechanism of a secondary noncontact ACL injury. Lastly, future research should include other patient‐reported outcome measures to assess other fear‐related constructs, such as Anterior Cruciate Ligament Return to Sport after Injury scale (ACL‐RSI).

## 7 CONCLUSION

The ACLR group had lower brain activity in the right ventrolateral prefrontal region relative to the uninjured control group during the AO‐DVJ paradigm. Secondly, in participants with ACLR, there were positive relationships between TSK‐11 scores and activity in brain areas typically engaged in fear and cognitive processes during the AO‐DVJ paradigm. Health care providers may need to consider the effects of kinesiophobia on neural control of movement during ACL rehabilitation.

## CONFLICT OF INTEREST

None of the authors have any conflicts of interest to report.

### PEER REVIEW

The peer review history for this article is available at https://publons.com/publon/10.1002/brb3.2879.

## Supporting information

Figure S1. Brain activity comparison between those with (Blue cluster) and without two high fear uninjured control and four low fear ACLR participants (Red cluster)

Table S1. Movement imagery questionnaire subscale

## Data Availability

The data that support the findings of this study are available from the corresponding author upon reasonable request.
