# Supplementary figures and images for: The relationship between drop vertical jump action‐observation brain activity and kinesiophobia after anterior cruciate ligament reconstruction: A cross‐sectional fMRI study
Source: Brain Behav. 2023 Jan 5;13(2):e2879. doi: 10.1002/brb3.2879 (PMC9927857; doi:10.1002/brb3.2879)

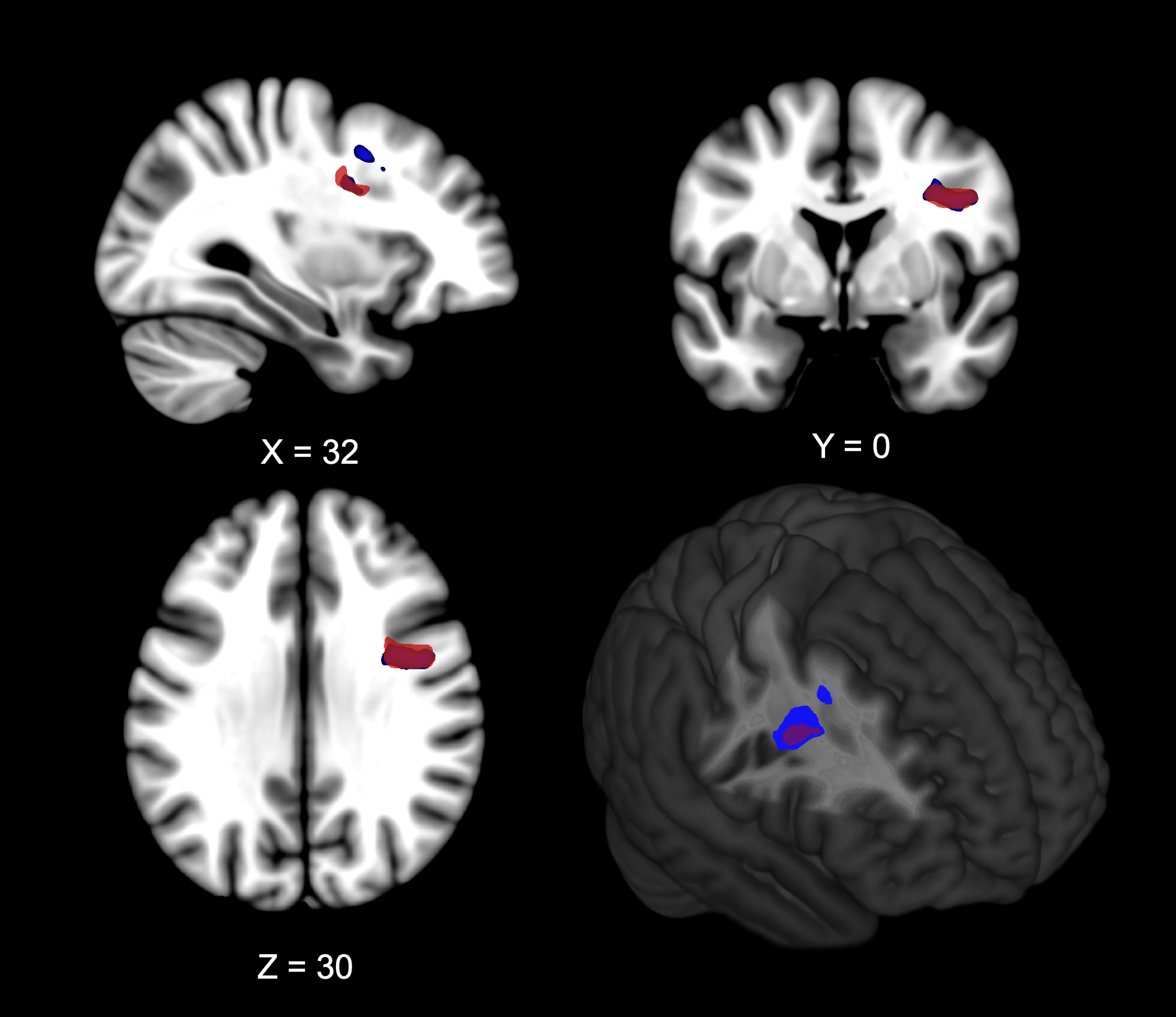

Supplement: Supplementary file 1 — Figure S1. Brain activity comparison between those with (Blue cluster) and without two high fear uninjured control and four low fear ACLR participants (Red cluster) [file BRB3-13-e2879-s001.png]
