# Supplementary material for: The relationship between drop vertical jump action‐observation brain activity and kinesiophobia after anterior cruciate ligament reconstruction: A cross‐sectional fMRI study
Source: Brain Behav. 2023 Jan 5;13(2):e2879. doi: 10.1002/brb3.2879 (PMC9927857; doi:10.1002/brb3.2879)
Supplement: Supplementary file 2 — Table S1. Movement imagery questionnaire subscale [file BRB3-13-e2879-s002.docx]

**Supplementary Table 1.** Movement Imagery Questionnaire Subscale.

|  | **ACL** | | **Control** | ***P*-value** | **Effect size** |
| --- | --- | --- | --- | --- | --- |
| Vertical Jump |  | |  |  |  |
| IVI | 5.54 ± 0.78 | | 5.54 ± 0.97 | 1.00 | 0.00 |
| EVI | 5.54 ± 0.97 | | 5.62 ± 1.19 | 0.86 | 0.07 |
| KI | 5.23 ± 1.17 | | 5.15 ± 0.80 | 0.85 | 0.08 |
| Hip Hike |  | |  |  |  |
| IVI | 6.00 ± 0.71 | | 6.23 ± 0.60 | 0.38 | 0.35 |
| EVI | 5.62 ± 1.12 | | 6.15 ± 0.99 | 0.21 | 0.51 |
| KI | 5.69 ± 0.85 | | 4.69 ± 1.49 | 0.05 | 0.82 |
| Bend Over | |  |  |  |  |
| IVI | 5.54 ± 1.27 | | 5.69 ± 1.18 | 0.75 | 0.13 |
| EVI | 5.85 ± 0.69 | | 6.15 ± 0.99 | 0.37 | 0.36 |
| KI | 6.00 ± 1.00 | | 4.69 ± 1.65 | 0.02^*^ | 0.96 |
| Shoulder Horizontal Abduction | | |  |  |  |
| IVI | 5.69 ± 1.11 | | 6.08 ± 0.64 | 0.29 | 0.43 |
| EVI | 6.08 ± 0.64 | | 6.00 ± 1.08 | 0.83 | 0.09 |
| KI | 5.31 ± 1.25 | | 4.92 ±1.44 | 0.47 | 0.29 |

Motor Imagery Questionnaire subscales were reported as mean ± standard deviation.

* Significantly different *p*<0.05, Effect size: Cohen’s d, ACL: Anterior cruciate ligament reconstruction group, Control: Uninjured control group, IVI: Internal visual imagery, EVI: External visual imagery, KI: Kinesthetic imagery

**
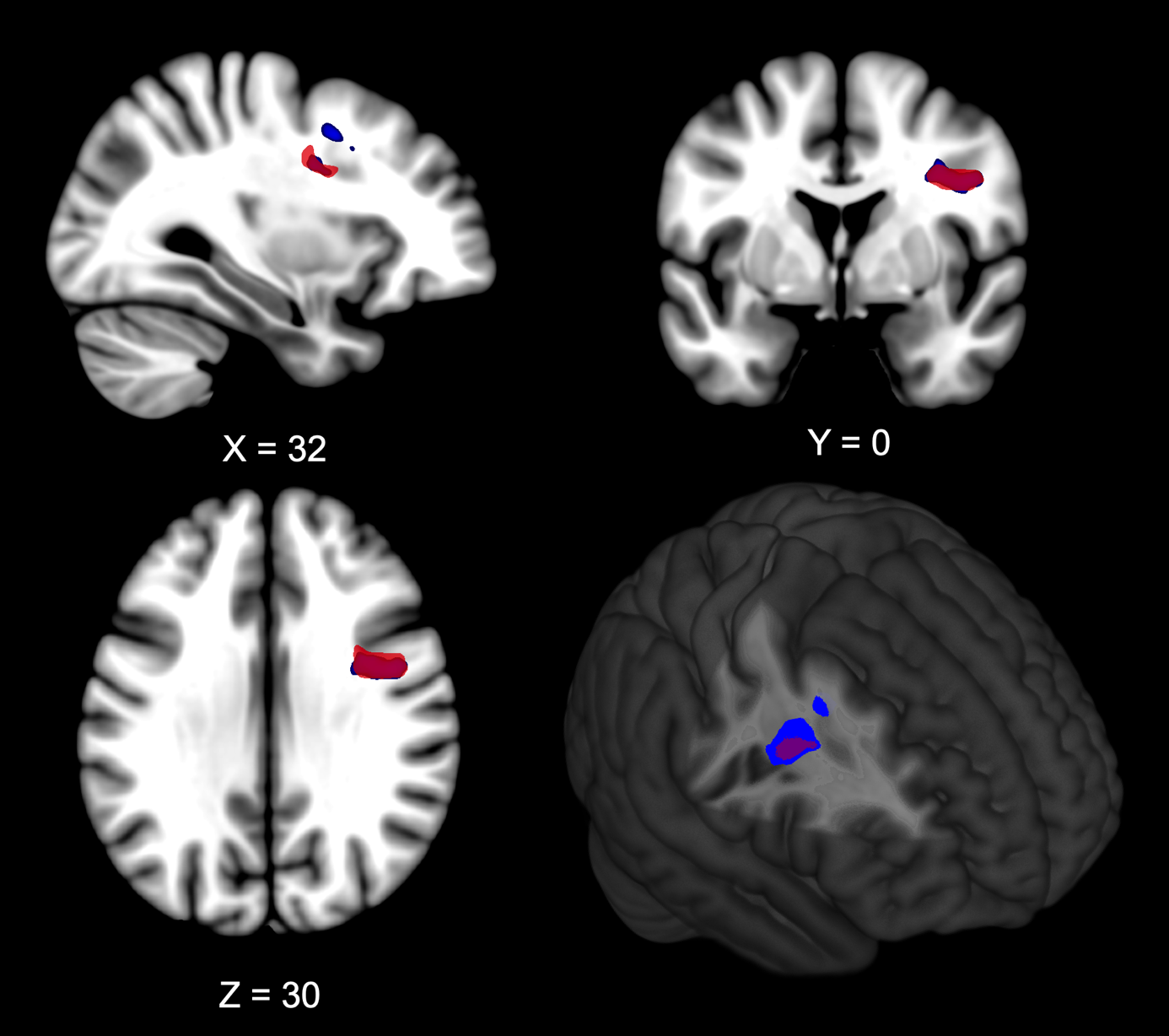
**

**Supplementary Figure 1.** Brain activity comparison between those with (Blue cluster) and without two high fear uninjured control and four low fear ACLR participants (Red cluster).
